# The Mallet

**Published:** 1870-05

**Authors:** 


### THE MALLET.

We have been trying so many different kinds of mallets that we have about come to the conclusion that we have no opinion upon the subject. While there were but two or three kinds, we thought we could decide something as to the best one; but there has been such a multitude of mallets—heavy, light, large, small, long, short, straight, crooked, round, oval, square; made of wood, horn, rubber, ivory, lead, tin, brass, copper, zinc, silver, iron, steel and

*potmetal*, that we are quite bewildered, and don't know what to say. Almost everything we pick up is a mallet; or at least the first thought is, that's a mallet.

We have become so confused on this subject, that if we use a drill we take a mallet; and if it's a chisel, we must use a mallet, and an excavator refuses to perform duty if the mallet is not on hand.

When we go to operate, we don't venture to make a selection ot a mallet, but shut our eyes, reach out and take the first one that presents, and each time we conclude that the one in hand is a pretty good thing in its way. And we have not yet been rash enough to venture an expression of dislike for any of them since the early part of the disease. We did then, once or twice, suggest that the ten-ounce mallet was not the best, and A said we did not know what we were talking about, and we acquiesced. We once ventured that the ounce mallet might be more efficient if a little heavier. B said he was surprised to find us so ignorant about the movement of things, and we immediately replied "yes," but it took several minutes for him to be fully convinced that we understood all about it, but our reply brought him finally.

We once expressed some doubts as to the superiority of the lead mallet. C said we were a fool on that subject, and he proved it too, as he thought, right in our face and teeth. We pocketed the insult.

These examples convey but a faint idea of the difficulty we have had to keep peace with our friends. We are determined henceforth to be delighted with every mallet that comes along, regardless of its weight, size, or the material of which it is made.

We are now using a steel mallet, and while our face is toward St. Louis, we will say that it is the grandest mallet out; but it is open to one objection, that before the mallet fever broke out it would have been called a hammer, but it isn't a hammer *now*—it's a mallet!

